# Efficacy of Endoscopic Sutured Gastroplasty on Diabetes Mellitus Type 2—A Systematic Review

**DOI:** 10.1002/edm2.70057

**Published:** 2025-05-30

**Authors:** Fenna M. M. Beeren, Mitchell J. R. Harker, Arianne C. van Bon, Marcel J. M. Groenen, Peter D. Siersema

**Affiliations:** ^1^ Department of Gastroenterology and Hepatology Rijnstate Hospital Arnhem the Netherlands; ^2^ Department of Gastroenterology and Hepatology Radboud University Medical Center Nijmegen the Netherlands; ^3^ Department of Bariatric Surgery Rijnstate Hospital Arnhem the Netherlands; ^4^ Department of Internal Medicine Rijnstate Hospital Arnhem the Netherlands; ^5^ Department of Gastroenterology and Hepatology Erasmus MC University Medical Center Rotterdam the Netherlands

**Keywords:** diabetes mellitus type 2, endoscopic gastrointestinal surgery, weight loss

## Abstract

**Introduction:**

Endoscopic sutured gastroplasty (ESG) is one of the currently available endoscopic techniques that mimics bariatric surgery. Although the efficacy of ESG on weight loss has repeatedly been demonstrated, its effect on diabetes mellitus type 2 (T2DM) related outcomes is not yet fully clear.

**Methods:**

We conducted a systematic review summarising the efficacy of ESG on T2DM. Therefore, PubMed, Embase and Cochrane library were searched for studies evaluating the effect of ESG on T2DM. Both prospective and retrospective studies, published in the English language, were included. Two reviewers independently screened all studies. The primary objectives were T2DM remission rate, decrease in glucose‐lowering medication and decrease in HbA1c.

**Results:**

A total of 16 studies including 760 patients with (pre)diabetes were included. Overall, T2DM‐related outcomes after ESG improved in 303 of 599 patients (50.6%) (including tapering dose of insulin and/or improvement of HbA1c/fasting glucose). Remission rates of T2DM were reported in 6/11 studies and seen in 89 of 155 (57.0%) patients after 6–36 months.

**Conclusion:**

This systematic review on the efficacy of ESG on T2DM suggests that ESG is able to improve diabetes‐related outcomes in approximately half of treated patients, with T2DM remission rates in more than half of them.

## Graphical abstract

This systematic review included 16 studies analysing the effect of endoscopic sleeve gastroplasty (ESG) on type 2 diabetes mellitus (T2DM). ESG is able to improve diabetes‐related outcomes in approximately half of treated patients, with T2DM remission rates in more than half of them.

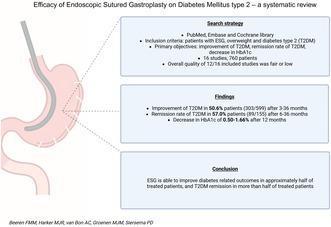

## 1 Introduction

Obesity (BMI > 30 kg/m^2^) is a major risk factor for various comorbidities, including cardiovascular disease, arthrosis, and several malignancies [1]. Moreover, each kilogram of weight gain is associated with a 4.5% increase in the risk of diabetes mellitus type 2 (T2DM) [2]. Conversely, weight loss has several beneficial aspects on T2DM, including increased insulin sensitivity, lower fasting insulin levels, and improved glycaemic control [3]. Interventions for weight loss include lifestyle interventions (e.g., dietary changes, physical activity), pharmacological treatment, bariatric surgery, and more recently, endoscopic bariatric procedures (e.g., endoscopic sutured gastroplasty (ESG), intra gastric balloon placement).

ESG is one of the available bariatric endoscopy techniques. It mimics a laparoscopic sleeve gastrectomy (LSG) by decreasing stomach size by approximating and stitching two plications of tissue to another on the stomach's greater curvature. This results in an approximately 70% reduction in stomach volume. For performing ESG, various endoscopic suturing devices are available, such as the Endomina (Endotools Therapeutics S.A., Gosselies, Belgium) and the Overstitch Endoscopic Suturing System (Apollo Endosurgery, Austin Texas, USA) [4]. The effectiveness of ESG on weight loss has been demonstrated in several studies. Hedjoudje et al. performed a meta‐analysis to evaluate the efficacy of ESG on weight loss, including 1772 patients (8 studies) with a mean BMI at baseline between 33.3 and 43.0 kg/m^2^. Mean % excessive weight loss (%EWL) was found to be 61.8% (95% CI 54.7%–68.9%) after 12 months [4].

In addition to the weight loss effect, bariatric surgery has been shown to improve diabetes‐related outcomes. Meta‐analyses have consistently demonstrated higher diabetes remission rates in patients with bariatric surgery‐induced weight loss, compared to those achieving similar weight loss without surgery [5, 6]. However, the exact mechanism by which ESG affects metabolic outcomes remains unclear, although several hypotheses have been proposed. First, cellular alterations may induce a decreased chronic inflammatory state leading to improved glycemic control. Second, a similar effect on the chronic inflammatory state may occur due to changes in the microbiome, mainly in lipopolysaccharides (LPS) from gram‐negative bacteria. Third, histological changes in organs involved in glucose metabolism due to weight loss may have an impact on insulin sensitivity [6]. The prevailing hypothesis suggests, however, that alterations in hormone secretion enhance postprandial glucose metabolism. These hormonal changes differ between ESG and LSG, particularly with regard to gut peptides such as GLP‐1 and ghrelin. ESG has been shown to induce minimal changes in hormones, whereas LSG is associated with a significant decrease in ghrelin and a substantial increase in GLP‐1 levels [7]. Therefore, the effect of ESG on glycemic control may potentially differ from that of the better‐studied LSG.

Endoscopic bariatric procedures provide a less invasive alternative to surgical bariatric procedures with a more favourable safety profile [8]. However, the effects of ESG on diabetes have remained insufficiently studied. It is not known whether the above‐mentioned mechanisms underlying diabetes remission after bariatric surgery also apply to ESG. For instance, ESG does not result in anatomical alterations in the small intestine as a gastric bypass does, but it may accelerate gastric emptying, leading to faster transit of food to the duodenum. This accelerated delivery of nutrients can elicit an altered hormonal response. This mechanism, in conjunction with weight loss, may play a role in the glycemic effect of ESG in patients with T2DM [9]. In this systematic review, we analysed the effect of ESG on glycaemic control in patients with obesity and T2DM.

## 2 Methods

### 2.1 Search Strategy

This systematic review was prospectively registered in the PROSPERO international register (CRD42022366791) and conducted according to the Preferred Reporting Items for Systematic Reviews and Meta‐analyses (PRISMA) statement [10]. A systematic literature search using the electronic databases PubMed, Embase, and Cochrane Library was performed in April 2024. Search terms included keywords regarding the patient group (patients with overweight and T2DM), the intervention (ESG) and the outcomes of interest (diabetes related). The complete search strategy can be found in the Data S1.

### 2.2 Inclusion and Exclusion Criteria

Studies performed in human subjects published in the English literature were included in this systematic review. Inclusion criteria were ESG in patients with overweight (BMI ≥ 25 kg/m^2^); all or at least some of the included study population known with T2DM; and outcome data regarding T2DM (e.g., HbA1c or remission rate) available. Exclusion criteria were review or meta‐analysis, comments, and letters to the editor; studies using other endoscopic techniques than ESG (e.g., POSE 1.0 and POSE 2.0 (Primary Obesity Surgery Endoluminal)), and studies in a paediatric population (< 18 years).

### 2.3 Study Selection

After removing duplicates, two reviewers (M.J.R.H., F.M.M.B.) independently screened the studies to identify patients that met the inclusion and exclusion criteria. Screening was first done by title and abstract and, if applicable, followed by full text. Any disagreement was resolved by discussion with a third reviewer (M.J.M.G.). Additionally, for studies analysing an overlapping population, the most recent publication was included.

### 2.4 Data Extraction and Quality Assessment

Data on study design, patient characteristics, procedure characteristics, outcomes, and analysis were independently extracted by two reviewers using prespecified data collection forms. The two reviewers assessed the quality of each study using the National Institutes of Health (NIH) quality assessment tools specified for the study design of a particular study. Disagreements in quality assessment were resolved by discussion.

### 2.5 Statistical Analysis and Data Synthesis

Heterogeneity in the various study designs, outcome measures, and statistics precluded performing a quantitative meta‐analysis and therefore only descriptive data will be presented. The primary endpoints are diabetes‐related: T2DM remission rate, decrease in glucose‐lowering medication, and decrease in HbA1c. Secondary endpoints included weight loss‐related outcomes, such as mean weight loss, %EWL, and % total weight loss (%TWL).

## 3 Results

### 3.1 Study Selection

**FIGURE 1:**
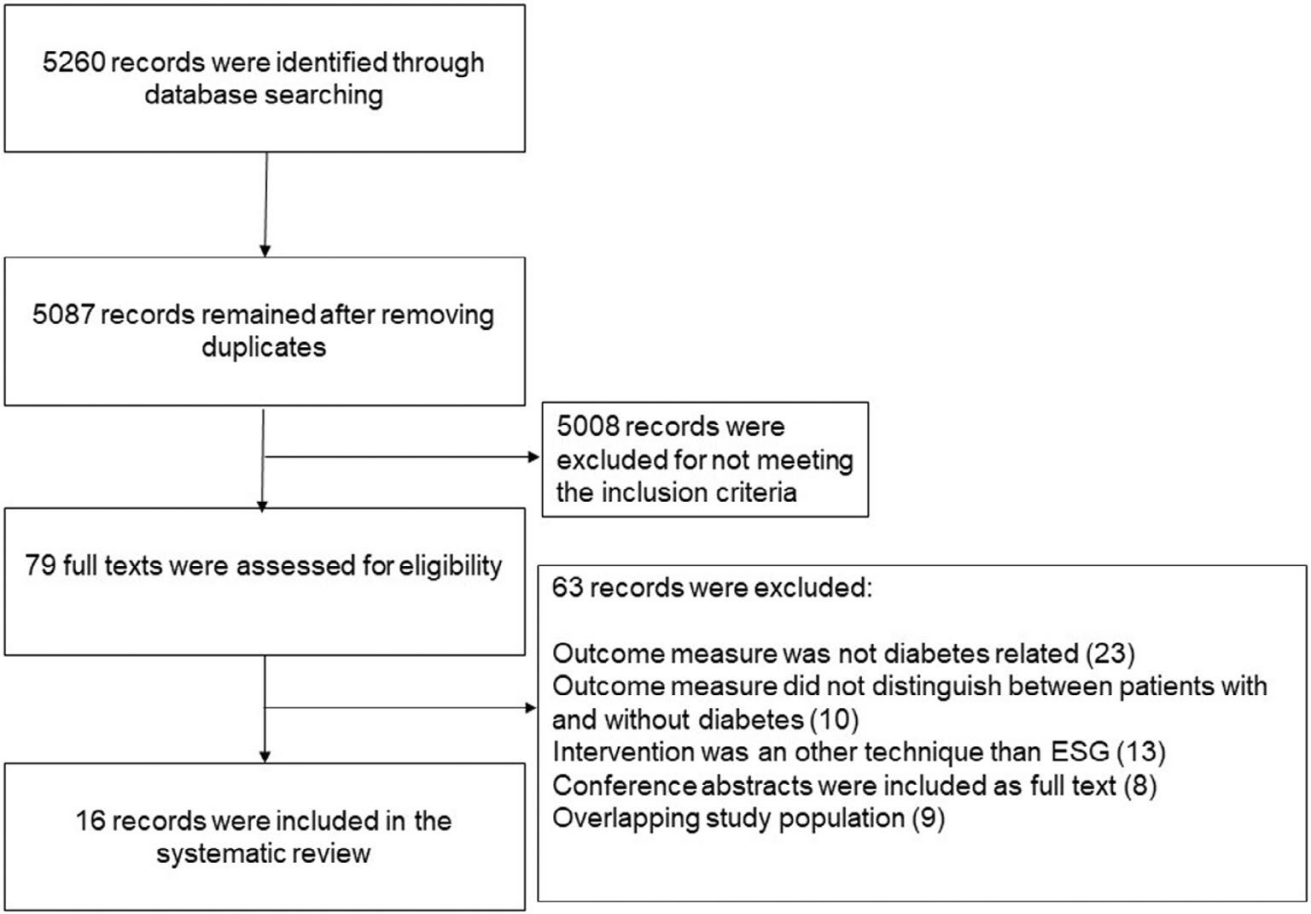
Flow diagram of the selection process.

The screening and selection process is shown in Figure 1. A total of 5260 studies were identified, of which 5087 remained after removing duplicates. After screening for title and abstract, 5008 records did not meet the inclusion criteria. Among the 79 studies that were screened as full text, 16 met all inclusion criteria, including four congress abstracts [11, 12, 13, 14, 15, 16, 17, 18, 19, 20, 21, 22, 23, 24, 25, 26].

### 3.2 Study Characteristics

The characteristics of the included studies are presented in Table 1. Of the 16 included studies, two were randomised controlled trials (RCT's) [11, 18], three non‐randomised comparisons [16, 17, 21], five retrospective observational studies [14, 23, 24, 25, 26], five prospective observational studies [12, 15, 19, 20, 22] and one a matched case control study [13]. In total, 4942 participants were included in these studies, of which 760 were known with (pre)T2DM with a baseline BMI of ≥ 25.5 kg/m^2^.

**TABLE 1:** Study and baseline characteristics.

First author	Abu Dayyeh [11]	Alexandre [12]	Alqahtani [13]	Asokkumar [14]	Bhandari [15]	Espinet‐Coll [16]	Familiari [17]	Huberty [18]	Jagtap [19]	Leccesi [20]	Li [21]	Maselli [22]	Matteo [23]	Reja [24]	Sarkar [25]	Westerveld [26]
Year of publication	2022 (CA)	2023	2022	2023	2022	2019	2011	2020	2021	2021	2021	2023	2021 (CA)	2021 (CA)	2022	2022 (CA)
Study design	RCT	Prospective observational study	Case control study	Retrospective observational study	Prospective observational study	Prospective intervention study	Prospective intervention study	RCT	Prospective overvation study	Prospective observational study	Prospective intervention study	Prospective observational study	Retrospective observational study	Retrospective observational study	Retrospective observational study	Retrospective observational study
No. of patients with diabetes/prediabetes/total	20/25/77	26/−/99	112/−/3018	3/−/15	271/−/612	3/−/15	14/−/67	4/−/49	10/13/26	5/−/50	8/−/24	72/−/387	1/−/65	67/−/92	6/−/91	−/103/255
Follow‐up (months)	24	12	36	12	24	12	12	18	12	12	12	12	12	3	12	60
Mean age (years)	NR	45 ± 12.7	33.8 ± 9.6	42 ± 8.5	40.7 ± 12.66	45.9 ± 13.8	41.0 ± 9.7	37.6 ± 9.9	41.5 ± 9.58	40.3 ± 8.9	55.6 ± 9.2	42.9 ± 9.4	46.3 ± 9.4	43.2 ± 11.4	39.7 ± 11.6	45.5 ± 11.9
Mean BMI (kg/m^2^)	NR	42.7 ± 7.8	32.5 ± 3.1	34.9 ± 4.4	34.3 ± 5.05	39.8 ± 6.78	41.5 ± 3.6	34.8 ± 2.7	36.6 ± 5.07	42.16 ± 3.8	49.9 ± 14.4	44.8 ± 4.7	34.3 ± 1.4	39.7 ± 11.6	38.7 ± NR	38.6 ± NR

Abbreviations: BMI, body mass index; CA, conference abstract; NR, not reported; RCT: randomised controlled trial.

### 3.3 Primary Endpoints

The included studies examined a variety of diabetes‐related endpoints (Table 2). Overall, diabetes‐related outcomes improved in 303/599 participants (50.6%) [11, 12, 13, 15, 16, 18, 19, 20, 21, 22, 23, 24].

**TABLE 2:** Primary endpoints.

First author	Abu Dayyeh [11]	Alexandre [12]	Alqahtani [13]	Asokkumar [14]	Bhandari [15]	Espinet‐Coll [16]	Familiari [17]	Huberty [18]	Jagtap [19]	Leccesi [20]	Li [21]	Maselli [22]	Matteo [23]	Reja [24]	Sarkar [25]	Westerveld [26]
Number of patients with improvement of T2DM—*n* (%)	14 (70)—12 m				121 (51.2)—24 m						7 (87.5)—12 m	44 (61.7)—36 m				
Number of patients with remission of T2DM—*n* (%)		8 (30.8)—6 m 9 (32.7)—12 m	72 (64.3)—36 m			1 (33.3)—12 m				5 (100)—12 m	1 (12.5)—12 m		1 (100)—6 m			
Number of patients that tapered or decreased dose of insulin—*n* (%)														23 (34.3)—3 m		
Number of patients that could stop all medication—*n* (%)								2 (50)—12 m	3 (30)—12 m							
Improvement of fasting glucose (mg/L)		0.2–6 m 0.14–12 m														
Improvement of HbA1c (%)				1.66–12 m			1.30–12 m		1.51–6 m 1.49–12 m						2.0–6 m 0.5–12 m	
Improvement of HbA1c (%) including prediabetes patients	1.65–12 m															0.70–12 m 0.30–36 m 0.60–60 m

Abbreviations: m, months; T2DM, diabetes mellitus type.

**FIGURE 2:**
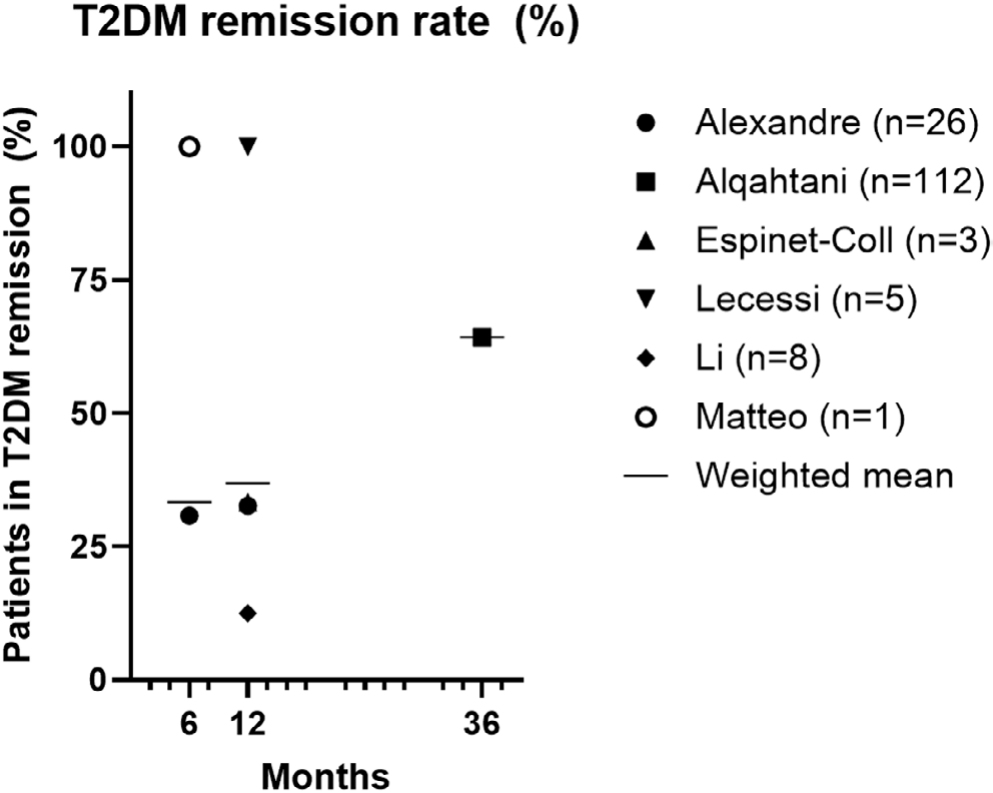
Diabetes mellitus type 2 remission rate (%). T2DM, diabetes mellitus type 2.

Remission rates of T2DM were reported in 6/11 studies and were observed in 89/155 patients (57.0%) after a follow‐up of 6–36 months (Figure 2) [12, 13, 16, 20, 21, 23]. Diabetes remission was defined differently in these six studies. In two of them, the Metabolic and Bariatric Surgery outcome reporting standards to define remission (HbA1c < 6.5%, fasting glucose < 100 mg/dL in the absence of antidiabetic medications) were used [13, 16], in one study a general definition for comorbidity remission, reduction of medication, and complete remission of relevant symptoms was used [21], while in the remaining three studies no remission rates were defined [12, 20, 23].

The first RCT by Abu Dayyeh et al. noticed an improvement of T2DM in the intervention group and standard medical care group of 70.0% and 26.7%, respectively (*p* = 0.002). Improvement of T2DM was not further defined [11]. The second RCT by Huberty et al. did not compare diabetes‐related outcomes between the two groups, since a cross‐over study design was used. The authors reported that overall, 2/4 patients with T2DM no longer used glucose‐lowering medication at 12 months after ESG [18].

**FIGURE 3:**
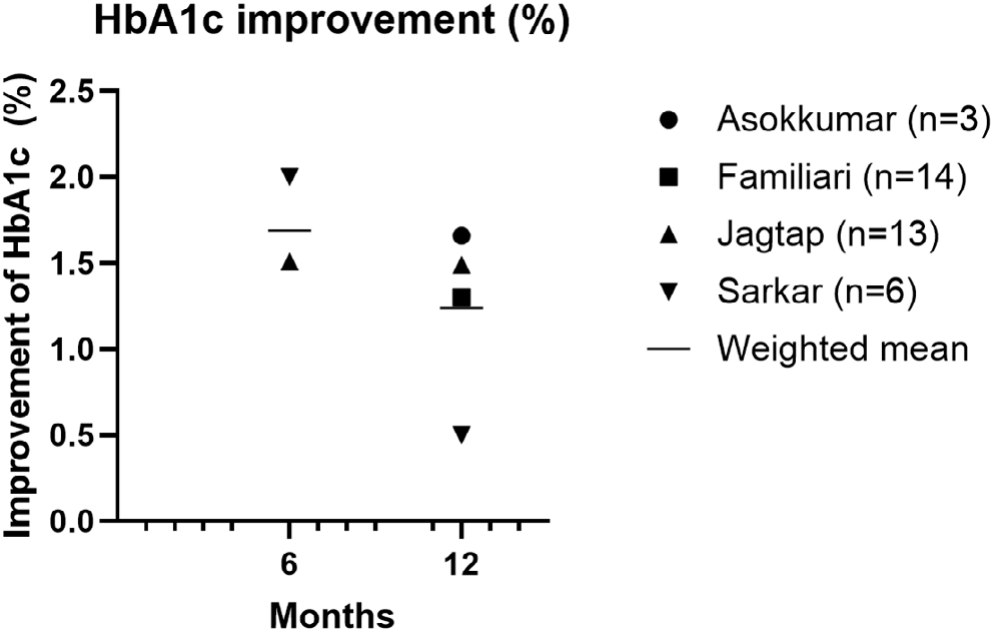
Improvement of HbA1c in patients with type 2 diabetes (%).

In two studies, 30%–50% of patients were able to stop all glucose lowering medication after 12 months [18, 19], and in one study 34.4% of patients could taper or reduce insulin doses after 3 months [24]. Finally, a decrease in HbA1c of 1.30%–1.66% at 12 months was reported in three studies (Figure 3) [11, 12, 17, 19, 25], while one study showed a decrease in HbA1c of 0.50 25 [25].

### 3.4 Secondary Endpoints

**FIGURE 4:**
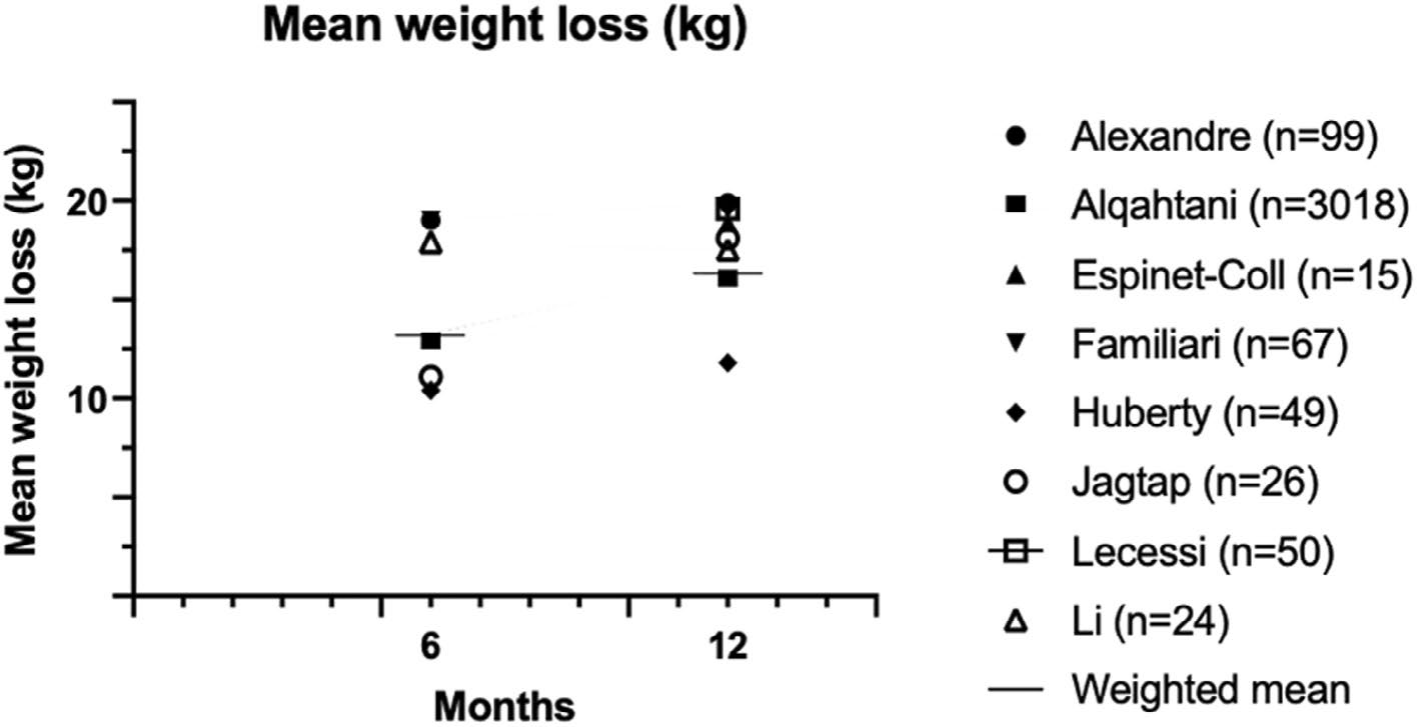
Mean weight loss in kilograms (kg).

**FIGURE 5:**
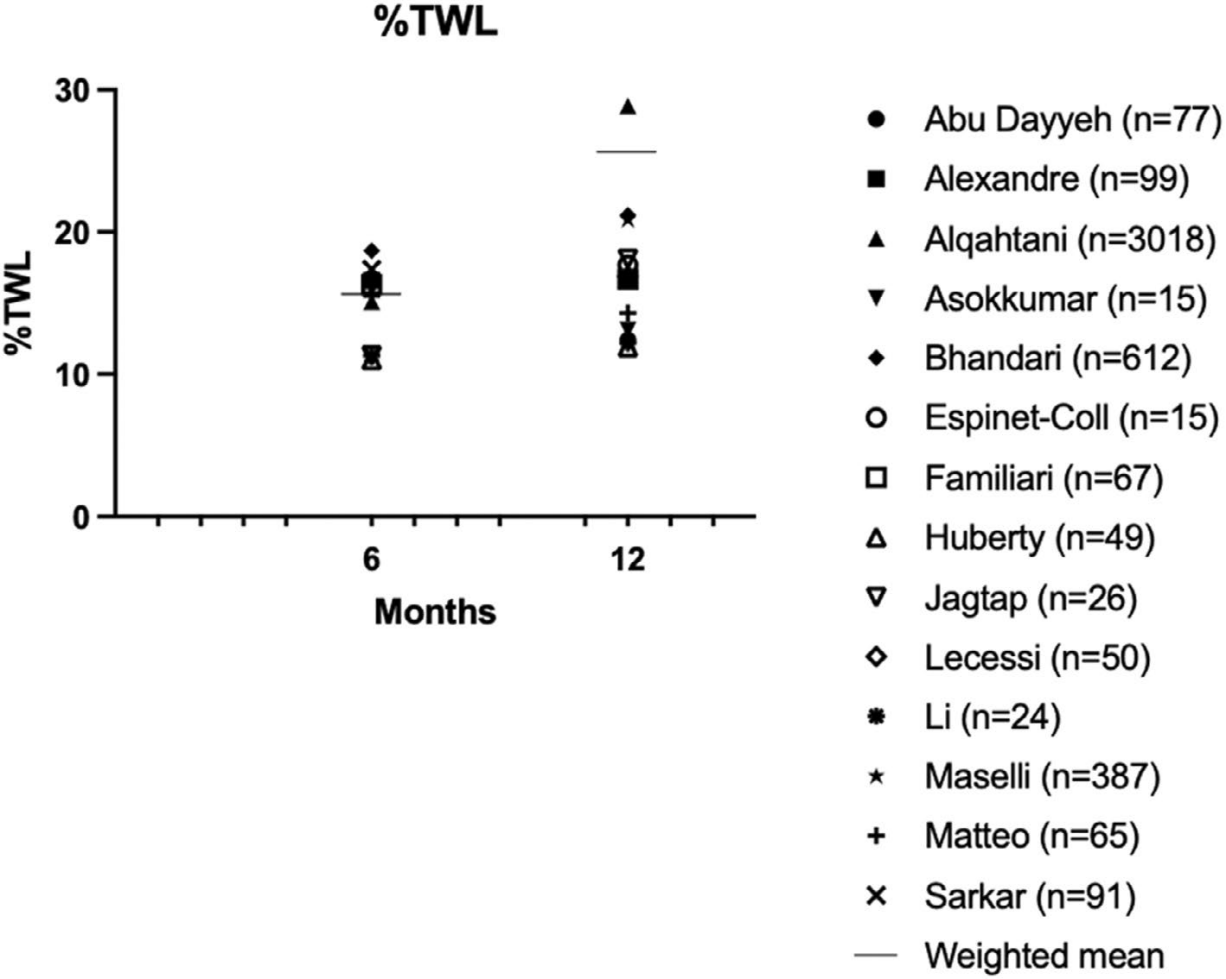
%Total weight loss (%TWL).

**FIGURE 6:**
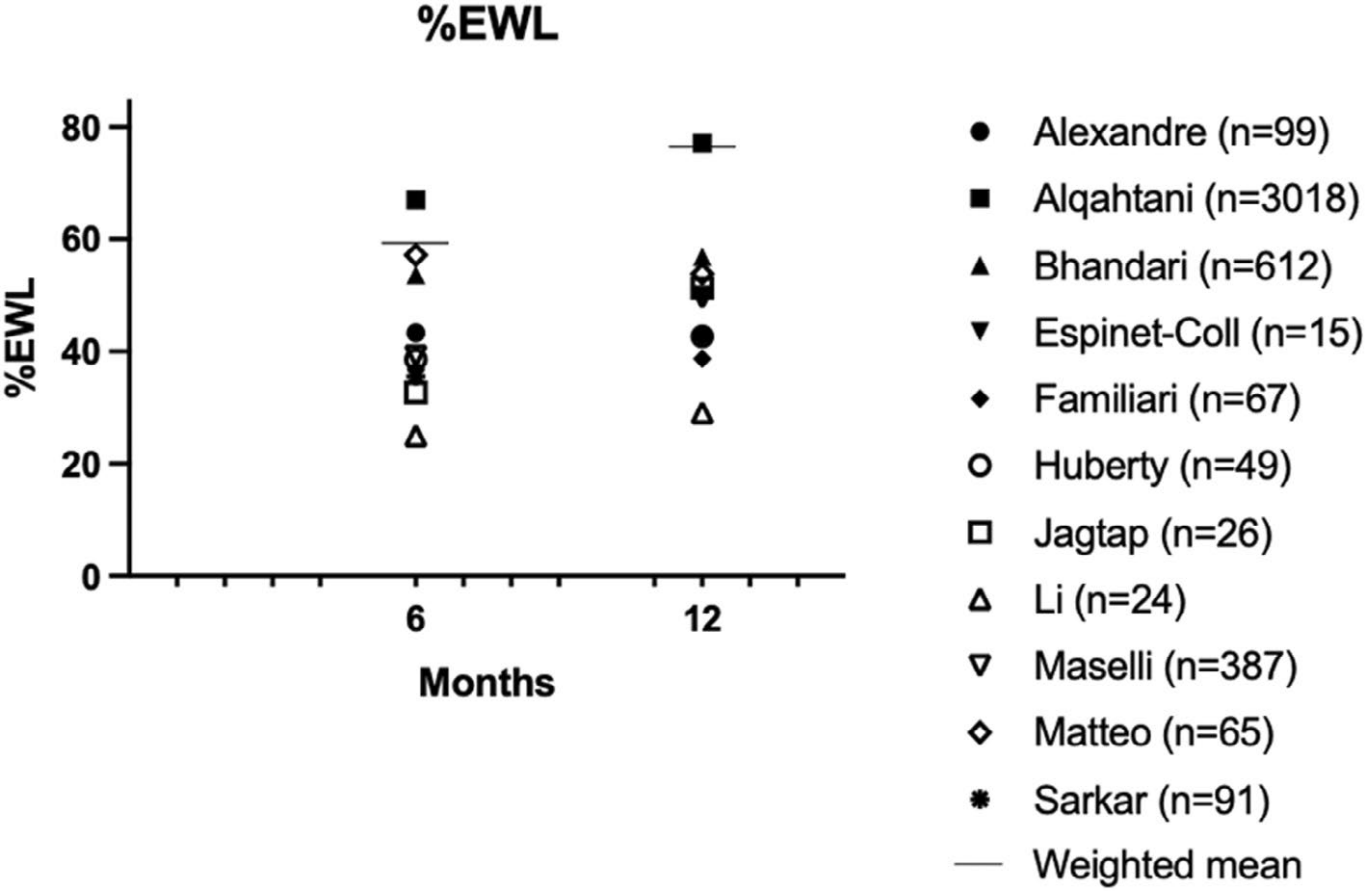
%Excess weight loss (%EWL).

Secondary outcomes are presented in Figures 4, 5, 6. Fifteen studies presented mean weight loss, %EWL and/or %TWL outcomes at 6–12 months [11, 12, 13, 14, 15, 16, 17, 18, 19, 20, 21, 22, 23, 24, 25] In ten studies, %EWL ranged from 29.1%–77.1% at 12 months, with a weighted mean of 76.4% [12, 13, 15, 16, 17, 18, 19, 21, 22, 23]. In thirteen studies %TWL ranged from 11.9%–28.9% at 12 months (weighted mean 25.7%) [11, 12, 13, 14, 15, 16, 17, 18, 19, 20, 21, 22, 23]. The longest follow‐up was 48 months and showed a %EWL of 49.3% and %TWL of 18.19% [15].

#### 3.4.1 Quality Assessment

Overall, four of sixteen studies were classified as good quality studies [11, 12, 20, 22], eight as fair [13, 14, 15, 17, 18, 19, 25, 26] and four as poor [16, 21, 23, 24] (Table 3). Limitations of the studies were mostly related to high numbers of patients lost to follow‐up and lack of reporting data (e.g., power calculation and participation rate of eligible patients). The detailed quality assessment can be found in the Data S1.

**TABLE 3:** Overall quality assessment.

First author	Overall quality assessment
Abu Dayyeh [11]	Good
Alexandre [12]	Good
Alqahtani [13]	Fair
Asokkumar [14]	Fair
Bhandari [15]	Fair
Espinet‐Coll [16]	Poor
Familiari [17]	Fair
Huberty [18]	Fair
Jagtap [19]	Fair
Leccesi [20]	Good
Li [21]	Poor
Maselli [22]	Good
Matteo [23]	Poor
Reja [24]	Poor
Sarkar [25]	Fair
Westerveld [26]	Fair

## 4 Discussion

### 4.1 Diabetes Mellitus Type 2

The long‐term remission rate for T2DM following ESG in this review was 57.0%, which is comparable to the remission rate of 60.8% observed in patients with T2DM who underwent LSG [27]. This suggests that ESG and LSG offer similar metabolic benefits. Furthermore, 303 out of 599 (50.6%) patients showed improvement in one or more T2DM‐related outcomes, such as remission or reduction of medication use. However, not all outcomes were reported in all included studies.

In contrast, uniform improvement in T2DM was observed in all LSG patients (20/20), suggesting potentially higher efficacy [28]. However, comparing results of all treatments is challenging due to the fact that no direct comparative studies between diabetes‐related outcomes in ESG and LSG have been performed. Further research, including RCTs, is needed to directly compare the efficacy and safety of ESG and LSG for T2DM management.

The hormonal response to ESG and LSG may explain differences in metabolic outcomes, particularly regarding ghrelin and GLP1 regulation. Both procedures enhance GLP‐1 secretion through accelerated gastric emptying and increased intestinal transit, leading to improved insulin sensitivity and glucose control [29]. However, the effects of ESG and LSG on ghrelin differ significantly. Ghrelin, primarily secreted by the gastric fundus and body, stimulates appetite, increases gastric emptying, and influences glucose metabolism during periods of starvation [30]. In a LSG, the removal of the gastric fundus results in a significant reduction in ghrelin levels, which likely contributes to reduced appetite and greater weight loss [31]. In contrast, an ESG preserves the fundus, allowing continued ghrelin secretion, which may partially explain the lower degree of weight loss observed compared to LSG [31]. While both ESG and LSG influence GLP‐1 secretion similarly, the distinct effects on ghrelin highlight the need for further research to determine how hormonal modulation impacts long‐term metabolic outcomes. Future studies should investigate whether targeted interventions, such as pharmacotherapy or coagulation of the fundus to suppress ghrelin or enhance GLP‐1 activity, could optimise ESG outcomes and bridge the gap in efficacy compared to LSG.

An increasing number of patients with type 1 diabetes mellitus (T1DM) are also overweight [32]. Bariatric surgery, mainly Roux‐en‐Y gastric bypass, has been shown to result in a reduction of HbA1c and insulin dose in patients with T1DM [33]. Since hormonal shifts are not very different in T1DM and T2DM, it is expected that the effect of ESG on T2DM will be similar in T1DM; however, ESG has so far only been anecdotally performed in T1DM patients [33].

### 4.2 Weight Loss

The %TWL observed in our systematic review for ESG was 11.9%–28.9% after 12 months, which is consistent with a previous meta‐analysis that summarised results of ESG in overweight and obese patients showing an %TWL of 16.5% at 12 months and 17.1% at 18–24 months [4]. The same findings were found for %EWL: 29.1–77.1% after 12 months in our study compared to 66.9% in the meta‐analysis of Hedjoudje et al. [4] This %EWL meets the efficacy threshold of 25% at 12 months, as established by the American Society for Gastrointestinal Endoscopy (ASGE).

LSG results in a mean %EWL of 71.2% after 12 months [34]. This is in the upper range of reported %EWL after ESG in our study. It is important to note that baseline BMI often differs between patients that have undergone LSG or ESG in studies. For LSG, this is generally a BMI > 35 kg/m^2^, but for ESG this is frequently lower, BMI > 25 kg/m^2^ (this review). As mentioned earlier, the effect of continued ghrelin secretion after ESG, and not after LSG, could also be an explanation for a seemingly lesser decrease in weight after ESG.

### 4.3 LSG or ESG

ESG's safety profile is superior compared to LSG. The pooled adverse event rate for LSG has been reported to be 11.8%, whereas for ESG it was just 2.9% [35]. This is mainly due to post‐operative gastroesophageal reflux disease (GERD) and bleeding events after LSG, which occur less often after ESG, 5.8% vs. 0.4% and 2.6% vs. 1.1%, respectively [8, 35] Although the lower adverse event rates favour ESG, the short follow‐up in studies conducted so far remains a limitation. ESG results are mostly based on approximately 12‐month follow‐up. Therefore, ESG was often preferably seen as an option for short‐term weight loss e.g., as a bridge to bariatric surgery. The first long‐term study results (5 years) however have been published and show promising results on both weight loss and HbA1c, with a %EWL of 45.3% (95% CI, 32.9–57.7; *p* < 0.001) after 5 years and an improvement in HbA1c in patients with diabetes from 6.4% to 5.8% (*p* = 0.01) [26, 36]. Combining these long‐term results with the lower adverse event rates, we suggest considering ESG as a primary treatment for patients with obesity and DM2 as well.

### 4.4 GLP‐1 Receptor Agonists or ESG

In addition to bariatric techniques, GLP‐1 receptor agonists are increasingly being used as a treatment for diabetes and obesity. However, after 68 weeks of treatment, 67.5% (257/381) of patients still have an HbA1c ≥ 6.5%, whereas only 28.6% were able to reduce their concurrent diabetes medication [37]. These results indicate that GLP‐1 receptor agonists improve glycemic control but do not lead to diabetes remission. Consequently, they appear to be less effective in achieving remission compared to ESG and LSG, which have remission rates of 57% and 60.8% respectively. Similarly, while GLP‐1 receptor agonists are effective for weight loss, they are not as effective as ESG. After 68 weeks, %TWL with GLP‐1 receptor agonists is 9.64% to 14.9%, whereas ESG achieves %TWL ranging from 11.9% to 28.9% after 52 weeks, as reported in this review [37, 38]. However, GLP‐1 receptor agonists also may have advantages over ESG as they are less invasive, resulting in a lower risk of endoscopic complications such as gastric perforation or complications related to sedation. Additionally, GLP‐1 receptor agonists serve as a direct pharmacotherapeutic treatment for diabetes, whereas ESG only has an indirect effect on diabetes.

### 4.5 Strengths and Limitations

This is the first systematic review that summarises the evidence of ESG on DM2. We included a variety of study designs and publication types. Moreover, various T2DM‐related outcomes are summarised within this review. We believe that this approach allows us to draw some conclusions on the effectiveness of ESG on T2DM. In addition, several study designs were used in the different studies. The NIH quality assessment tool rated three‐quarters of the included studies as fair (8/16) or poor (4/16) methodology quality, which compromises the reliability of the presented data. This highlights an area for improvement in future research on ESG.

This systematic review comes also with some limitations, of which the main one was the heterogeneity between studies in several different aspects, such as study design, study outcome definition, and study intervention. There were no studies, especially no RCTs, that used diabetes‐related outcomes as a primary endpoint. Second, no consistency was seen in inclusion criteria. For example, some studies included patients with a maximum BMI of 35 kg/m^2^ [13, 16, 23], while other studies included patients with a BMI > 35 kg/m^2^ or even > 50 kg/m^2^ [17, 20, 21]. Third, study interventions were also not uniformly performed. For example, only ESG was performed in nine studies [12, 15, 17, 19, 20, 21, 23, 24, 26], while in seven other studies ESG was combined with lifestyle and diet guidance [11, 13, 14, 16, 18, 22, 25]. Nevertheless, an unambiguous success of ESG was observed across the entire range of diabetes‐related endpoints, BMIs, and interventions. Fourth, several studies used descriptive statistics to provide information on comorbidities, without performing a statistical analysis. This made it not possible to perform a meta‐analysis. Last, the reason that the overall quality of three‐quarters of included studies was low or fair (see above) was mostly due to incomplete reporting. This heterogeneity in study designs, follow‐up durations, and definitions of T2DM remission posed challenges in synthesising consistent outcomes, thereby complicating the interpretation of the findings. This variability underscores the need for standardised reporting in future ESG trials.

## 5 Conclusion

This systematic review summarises the current evidence on the efficacy of ESG on T2DM, which suggests an improvement of diabetes‐related outcomes in more than 50% in persons with diabetes. Further studies are needed to strengthen this evidence.

## Author Contributions

The authors take full responsibility for this article.

## Conflicts of Interest

The authors declare no conflicts of interest.

## Supporting information


Data S1.

## Data Availability

The data that support the findings of this study are available from the corresponding author upon reasonable request.
