# Supplementary material for: Efficacy of Endoscopic Sutured Gastroplasty on Diabetes Mellitus Type 2—A Systematic Review
Source: Endocrinol Diabetes Metab. 2025 May 30;8(4):e70057. doi: 10.1002/edm2.70057 (PMC12124318; doi:10.1002/edm2.70057)
Supplement: Supplementary file 1 — Data S1. [file EDM2-8-e70057-s001.docx]

**Supplementary file 1 – Search strategy**

**Pubmed:**

(((endoscop*[tiab] OR endoluminal[tiab]) AND (sutur*[tiab] OR sleeve*[tiab] OR gastroplast*[tiab] OR gastrectom*[tiab] OR bariatr*[tiab] OR plicat* [tiab])) OR ESG [tiab] OR Endomina [tiab] OR POSE [tiab] OR overstitch [tiab] OR apollo [tiab] OR endosleeve* [tiab] OR endobariatr*[tiab] OR (transoral[tiab] AND gastroplast*[tiab]) OR (endoscop*[tiab] AND gastric reduction [tiab]) OR (metabolic endosc*[tiab]))

OR

((bariatric surgery[Mesh:noexp] OR gastroplasty[Mesh]) AND (endoscopy[Mesh] OR gastroscopy[Mesh]))

AND

(((diabet* [tiab] AND ((type 2 [tiab]) OR (type II [tiab]))) OR ((fasting [tiab] OR blood [tiab]) AND glucose [tiab]) OR (glycemic control [tiab]) OR (glycaemic control [tiab]) OR (glycated hemoglobin A [tiab]) OR Hba1c [tiab] OR ((antidiabet* [tiab] OR anti-diabet* [tiab] OR hypoglyc* [tiab]) AND agent* [tiab]) OR insulin [tiab] OR metformin [tiab] OR sulfonylureas [tiab] OR (DPP4 inhibitor* [tiab]) OR (GLP-1 receptor agonis* [tiab]) OR (diabetes medication [tiab]))

OR

((diabetes mellitus type 2[Mesh]) OR (blood glucose [Mesh]) OR (glycated hemoglobin A [Mesh]) OR (glycemic control [Mesh]) OR (Hypoglycemic Agents [Pharmacological Action])))

**Embase:**

(((endoscop*.ti,ab,kf. OR endoluminal.ti,ab,kf.) AND (sutur*.ti,ab,kf. OR sleeve*.ti,ab,kf. OR gastroplast*.ti,ab,kf. OR gastrectom*.ti,ab,kf. OR bariatr*.ti,ab,kf. OR plicat* .ti,ab,kf.)) OR ESG .ti,ab,kf. OR Endomina .ti,ab,kf. OR POSE .ti,ab,kf. OR overstitch .ti,ab,kf. OR apollo .ti,ab,kf. OR endosleeve*.ti,ab,kf. OR endobariatr*.ti,ab,kf. OR (transoral.ti,ab,kf. AND gastroplast*.ti,ab,kf.) OR (endoscop*.ti,ab,kf. AND gastric reduction.ti,ab,kf.) OR (metabolic endosc*.ti,ab,kf.))

OR

(((exp bariatric surgery/) OR gastroplasty/) AND ((gastrointestinal endoscopy/) OR gastroscopy/))

AND

(((diabetes.ti,ab,kf. AND (type 2.ti,ab,kf. OR type II.ti,ab,kf.)) OR ((fasting.ti,ab,kf. OR blood.ti,ab,kf.) AND glucose.ti,ab,kf.) OR (glycemic control.ti,ab,kf.) OR ((glycated hemoglobin A.ti,ab,kf. OR Hba1c.ti,ab,kf.)) OR ((antidiabet*.ti,ab,kf. OR hypoglyc*.ti,ab,kf.) AND agent*.ti,ab,kf.) OR insulin.ti,ab,kf. OR metformin.ti,ab,kf. OR sulfonylureas.ti,ab,kf. OR (DPP4 inhibitor.ti,ab,kf.) OR (GLP-1 receptor agonist.ti,ab,kf.) OR (diabetes medication.ti,ab,kf.)))

OR

((exp non insulin dependent diabetes mellitus/) OR (exp glucose blood level/) OR (glycemic control/) OR (exp haemoglobin A1c/) OR (exp antidiabetic agent/))

**Cochrane:**

((endoscop*:ti,ab OR endoluminal:ti,ab) AND (suture*:ti,ab OR sleeve*:ti,ab OR gastroplasty*:ti,ab OR gastrectomy*:ti,ab OR bariatr*:ti,ab OR plicat*:ti,ab))

OR

((ESG:ti,ab OR Endomina:ti,ab OR POSE:ti,ab OR overstitch:ti,ab OR apollo:ti,ab OR endosleeve*:ti,ab OR endobariatr*:ti,ab OR (transoral:ti,ab AND gastroplasty*:ti,ab) OR (endoscop*:ti,ab AND (gastric reduction):ti,ab) OR (metabolic endoscop*):ti,ab))

OR

(([mh gastroplasty] OR [mh “bariatric surgery”]) AND ([mh “Endoscopy, digestive system”] OR [mh gastroscopy]))

AND

((diabetes:ti,ab AND ((type 2):ti,ab OR (type II):ti,ab)) OR ((fasting:ti,ab OR blood:ti,ab) AND glucose:ti,ab) OR (glycemic control):ti,ab OR (glycaemic control):ti,ab OR Hba1c:ti,ab OR ((antidiabet*:ti,ab OR hypoglyc*:ti,ab) AND agent*:ti,ab) OR insulin:ti,ab OR metformin:ti,ab OR sulfonylureas:ti,ab OR (DPP4 inhibitor):ti,ab OR (GLP-1 receptor agonist):ti,ab)

OR

([mh “diabetes mellitus, type 2”] OR [mh “blood glucose”] OR [mh “glycated hemoglobin A”] OR [mh “glycemic control”] OR [mh “hypoglycemic agents”])

**Supplementary file 2 – Quality assessment included studies**

NR = not reported, NA = not applicable

**Controlled intervention study:**

|  | **Abu Dayyeh 2022** | **Espinet-Coll 2019** | **Huberty 2020** |
| --- | --- | --- | --- |
| 1. Was the study described as randomized, a randomized trial, a randomized clinical trial, or an RCT? | Yes | No | Yes |
| 2. Was the method of randomization adequate (i.e., use of randomly generated assignment)? | Yes | NA | Yes |
| 3. Was the treatment allocation concealed (so that assignments could not be predicted)? | Yes | NA | Yes |
| 4. Were study participants and providers blinded to treatment group assignment? | No | NA | No |
| 5. Were the people assessing the outcomes blinded to the participants' group assignments? | No | NR | No |
| 6. Were the groups similar at baseline on important characteristics that could affect outcomes (e.g., demographics, risk factors, co-morbid conditions)? | NR | Yes | No |
| 7. Was the overall drop-out rate from the study at endpoint 20% or lower of the number allocated to treatment? | Yes | Yes | Yes |
| 8. Was the differential drop-out rate (between treatment groups) at endpoint 15 percentage points or lower? | Yes | Yes | Yes |
| 9. Was there high adherence to the intervention protocols for each treatment group? | Yes | NA | NA |
| 10. Were other interventions avoided or similar in the groups (e.g., similar background treatments)? | NR | Yes | NR |
| 11. Were outcomes assessed using valid and reliable measures, implemented consistently across all study participants? | Yes | Yes | Yes |
| 12. Did the authors report that the sample size was sufficiently large to be able to detect a difference in the main outcome between groups with at least 80% power? | NR | No | Yes |
| 13. Were outcomes reported or subgroups analyzed prespecified (i.e., identified before analyses were conducted)? | Yes | Yes | Yes |
| 14. Were all randomized participants analyzed in the group to which they were originally assigned, i.e., did they use an intention-to-treat analysis? | Yes | Yes | NR |
| Overall quality | Good | Poor | Fair |

**Case-control study**

|  | **Alqahtani 2022** |
| --- | --- |
| 1. Was the research question or objective in this paper clearly stated and appropriate? | Yes |
| 2. Was the study population clearly specified and defined? | Yes |
| 3. Did the authors include a sample size justification? | No |
| 4. Were controls selected or recruited from the same or similar population that gave rise to the cases (including the same timeframe)? | Yes |
| 5. Were the definitions, inclusion and exclusion criteria, algorithms or processes used to identify or select cases and controls valid, reliable, and implemented consistently across all study participants? | Yes |
| 6. Were the cases clearly defined and differentiated from controls? | Yes |
| 7. If less than 100 percent of eligible cases and/or controls were selected for the study, were the cases and/or controls randomly selected from those eligible? | No |
| 8. Was there use of concurrent controls? | No |
| 9. Were the investigators able to confirm that the exposure/risk occurred prior to the development of the condition or event that defined a participant as a case? | NA |
| 10. Were the measures of exposure/risk clearly defined, valid, reliable, and implemented consistently (including the same time period) across all study participants? | Yes |
| 11. Were the assessors of exposure/risk blinded to the case or control status of participants? | No |
| 12. Were key potential confounding variables measured and adjusted statistically in the analyses? If matching was used, did the investigators account for matching during study analysis? | No |
| Overall quality | Fair |

**Before-after (pre-post) studies with no control group**

|  | **Asokkumar 2023** | **Familiari 2011** | **Li 2021** | **Maselli 2023** | **Sarkar 2022** |
| --- | --- | --- | --- | --- | --- |
| 1. Was the study question or objective clearly stated? | Yes | Yes | Yes | Yes | Yes |
| 2. Were eligibility/selection criteria for the study population prespecified and clearly described? | Yes | Yes | Yes | Yes | Yes |
| 3. Were the participants in the study representative of those who would be eligible for the test/service/intervention in the general or clinical population of interest? | Yes | Yes | Yes | Yes | Yes |
| 4. Were all eligible participants that met the prespecified entry criteria enrolled? | NR | NR | NR | NR | NR |
| 5. Was the sample size sufficiently large to provide confidence in the findings? | No | No | Yes | Yes | Yes |
| 6. Was the test/service/intervention clearly described and delivered consistently across the study population? | Yes | Yes | Yes | Yes | Yes |
| 7. Were the outcome measures prespecified, clearly defined, valid, reliable, and assessed consistently across all study participants? | Yes | Yes | Yes | Yes | Yes |
| 8. Were the people assessing the outcomes blinded to the participants' exposures/interventions? | No | No | No | No | No |
| 9. Was the loss to follow-up after baseline 20% or less? Were those lost to follow-up accounted for in the analysis? | Yes | No | No | Yes | No |
| 10. Did the statistical methods examine changes in outcome measures from before to after the intervention? Were statistical tests done that provided p values for the pre-to-post changes? | Yes | Yes | No | Yes | Yes |
| 11. Were outcome measures of interest taken multiple times before the intervention and multiple times after the intervention (i.e., did they use an interrupted time-series design)? | Yes | No | No | Yes | Yes |
| 12. If the intervention was conducted at a group level (e.g., a whole hospital, a community, etc.) did the statistical analysis take into account the use of individual-level data to determine effects at the group level? | NA | NA | NA | NA | NA |
| Overall quality | Fair | Fair | Poor | Good | Fair |

**Observational cohort and cross-sectional study**

|  | **Alexandre 2022** | **Bhandari 2023** | **Jagtap 2021** | **Lecessi 2021** | **Matteo 2021** |
| --- | --- | --- | --- | --- | --- |
| 1. Was the research question or objective in this paper clearly stated? | Yes | Yes | Yes | Yes | Yes |
| 2. Was the study population clearly specified and defined? | Yes | No | Yes | Yes | No |
| 3. Was the participation rate of eligible persons at least 50%? | NR | NR | NR | NR | NR |
| 4. Were all the subjects selected or recruited from the same or similar populations (including the same time period)? Were inclusion and exclusion criteria for being in the study prespecified and applied uniformly to all participants? | Yes | Yes | Yes | Yes | Yes |
| 5. Was a sample size justification, power description, or variance and effect estimates provided? | NR | No | No | No | NR |
| 6. For the analyses in this paper, were the exposure(s) of interest measured prior to the outcome(s) being measured? | Yes | Yes | Yes | Yes | Yes |
| 7. Was the timeframe sufficient so that one could reasonably expect to see an association between exposure and outcome if it existed? | Yes | Yes | Yes | Yes | Yes |
| 8. For exposures that can vary in amount or level, did the study examine different levels of the exposure as related to the outcome (e.g., categories of exposure, or exposure measured as continuous variable)? | NA | NA | NA | NA | NR |
| 9. Were the exposure measures (independent variables) clearly defined, valid, reliable, and implemented consistently across all study participants? | Yes | Yes | Yes | Yes | NR |
| 10. Was the exposure(s) assessed more than once over time? | NA | NA | NA | NA | NA |
| 11. Were the outcome measures (dependent variables) clearly defined, valid, reliable, and implemented consistently across all study participants? | Yes | Yes | Yes | Yes | Yes |
| 12. Were the outcome assessors blinded to the exposure status of participants? | No | NA | No | No | NR |
| 13. Was loss to follow-up after baseline 20% or less? | NR | Yes | NR | Yes | No |
| 14. Were key potential confounding variables measured and adjusted statistically for their impact on the relationship between exposure(s) and outcome(s)? | NR | No | No | Yes | NR |
| Overall quality | Fair | Fair | Fair | Good | Poor |

|  | **Reja 2021** | **Westerveld 2022** |
| --- | --- | --- |
| 1. Was the research question or objective in this paper clearly stated? | Yes | Yes |
| 2. Was the study population clearly specified and defined? | No | No |
| 3. Was the participation rate of eligible persons at least 50%? | NR | NR |
| 4. Were all the subjects selected or recruited from the same or similar populations (including the same time period)? Were inclusion and exclusion criteria for being in the study prespecified and applied uniformly to all participants? | Yes | No |
| 5. Was a sample size justification, power description, or variance and effect estimates provided? | NR | NR |
| 6. For the analyses in this paper, were the exposure(s) of interest measured prior to the outcome(s) being measured? | Yes | Yes |
| 7. Was the timeframe sufficient so that one could reasonably expect to see an association between exposure and outcome if it existed? | No | Yes |
| 8. For exposures that can vary in amount or level, did the study examine different levels of the exposure as related to the outcome (e.g., categories of exposure, or exposure measured as continuous variable)? | NA | NA |
| 9. Were the exposure measures (independent variables) clearly defined, valid, reliable, and implemented consistently across all study participants? | NR | Yes |
| 10. Was the exposure(s) assessed more than once over time? | NA | NA |
| 11. Were the outcome measures (dependent variables) clearly defined, valid, reliable, and implemented consistently across all study participants? | Yes | Yes |
| 12. Were the outcome assessors blinded to the exposure status of participants? | No | NR |
| 13. Was loss to follow-up after baseline 20% or less? | NR | NR |
| 14. Were key potential confounding variables measured and adjusted statistically for their impact on the relationship between exposure(s) and outcome(s)? | NR | NR |
| Overall quality | Poor | Fair |
